# Respiratory Ciliary Beat Frequency in COPD: Balancing Oxidative Stress and Pharmacological Treatment

**DOI:** 10.3390/antiox14111340

**Published:** 2025-11-06

**Authors:** Marta Joskova, Vladimira Sadlonova, Daniela Mokra, Ivan Kocan, Martina Sutovska, Karin Kackova, Sona Franova

**Affiliations:** 1 Department of Pharmacology, Jessenius Faculty of Medicine in Martin, Comenius University in Bratislava, SK-036 01 Martin, Slovakia; marta.joskova@uniba.sk (M.J.); martina.sutovska@uniba.sk (M.S.); kackova8@uniba.sk (K.K.); sona.franova@uniba.sk (S.F.); 2 Department of Microbiology and Immunology, Jessenius Faculty of Medicine in Martin, Comenius University in Bratislava, SK-036 01 Martin, Slovakia; 3 Department of Physiology, Jessenius Faculty of Medicine in Martin, Comenius University in Bratislava, SK-036 01 Martin, Slovakia; daniela.mokra@uniba.sk; 4 Clinic of Pneumology and Phthisiology, Jessenius Faculty of Medicine in Martin, Comenius University in Bratislava, SK-036 01 Martin, Slovakia; kocanivan@gmail.com

**Keywords:** COPD, oxidative stress, respiratory cilia, cilia kinetics, LABA, LAMA, PDE4, corticosteroids

## Abstract

In chronic obstructive pulmonary disease (COPD), dysregulated calcium homeostasis, oxidative stress, and mucus hypersecretion converge to suppress ciliary beat frequency (CBF), thereby compromising mucociliary clearance (MCC). These mechanisms are subject to pharmacological modulation. Long-acting muscarinic antagonists (LAMAs) exert direct cilia-stimulatory effects and may counteract pathogen-induced mucin overproduction without impairing clearance. Long-acting β_2_-agonists (LABAs) enhance ciliary activity through the cAMP–PKA–dynein (cyclic adenosine monophosphate–protein kinase A–dynein) signalling pathway. Inhaled corticosteroids (ICSs), although largely neutral on CBF, provide indirect protection by suppressing IL-13–driven inflammation. Phosphodiesterase (PDE)-4 inhibitors sustain intracellular cAMP and promote ciliary motility, though their clinical use remains limited by adverse effects. Emerging evidence suggests that dual and triple therapies may provide additive or synergistic benefits for preserving mucociliary function. Clinically, ex vivo CBF interpretation may be influenced by ongoing pharmacotherapy and tissue sampling site. Nasal brush samples may predominantly reflect systemic rather than inhaled therapy. Moreover, differences in PDE isoform expression between nasal and bronchial epithelium further complicate direct extrapolation of results. Rigorous patient stratification by treatment regimen is therefore essential to reconcile inconsistencies reported across studies. Ultimately, preservation of MCC in COPD depends on a delicate balance between oxidative stress and pharmacological modulation of ciliary function.

## 1. Introduction

Chronic obstructive pulmonary disease (COPD) is a complex global health issue characterised by persistent airway inflammation. It ranks among the top three causes of death worldwide, despite being both preventable and treatable. The primary treatment objectives focus on symptom reduction and minimising the risk of exacerbations. Clinically, acute respiratory events are manifested by dyspnoea, cough, and sputum production, which typically worsen over a period of fewer than 14 days.

Exacerbations of COPD are frequently linked to heightened local and systemic inflammation, often triggered by airway infections, environmental pollutants, and other harmful stimuli [1]. These episodes may partly result from impaired mucociliary clearance (MCC), which defends the airways by transporting mucus, particles, and toxins toward the oropharynx. This mechanism plays a critical role in preventing secondary complications through the coordinated beating of cilia.

When this mechanism fails, mucus accumulates, creating favourable conditions for microbial growth and inflammation. The resulting cascade contributes to airway obstruction, atelectasis, epithelial injury, airway remodelling, and, in severe cases, respiratory failure. Ultimately, such impairment diminishes responsiveness to standard therapy [2,3,4,5].

When MCC is compromised, the cough reflex can partly compensate for this dysfunction. This may indicate that diseases affecting only ciliary motion tend to be milder, whereas those involving both mucus and cilia are generally more severe. Such differences, as observed in primary ciliary dyskinesia (PCD) and cystic fibrosis, may lead to distinct clinical outcomes [5,6,7]. The most serious complications arise when both MCC and the cough reflex fail [8,9,10].

Given the central role of cilia in maintaining airway defence, pharmacological strategies that preserve or enhance ciliary function may offer therapeutic potential. In this context, the study was designed to examine how standard COPD therapy affects MCC, with particular emphasis on ciliary motor function under conditions of oxidative stress.

There is growing evidence for the cilia-stimulating effects of bronchodilators [11,12,13], which remain the cornerstone of COPD pharmacotherapy [1,14]. Nevertheless, some patients continue to experience frequent exacerbations or persistent productive cough despite optimal treatment and smoking cessation. This may reflect underlying impairment of ciliary function, including changes in ciliary signalling [15,16]. Enhancing airway clearance by supporting ciliary activity could therefore help prevent progression of airway inflammation, reduce the need for more aggressive therapy, and improve patients’ quality of life. To achieve this objective, the cilia must remain structurally intact, correctly oriented, and functionally active within the airway epithelium.

The impact of cigarette smoke on ciliary beat frequency (CBF) has been extensively investigated, as CBF represents a key determinant of MCC efficiency. In contrast, the pharmacodynamic effects of standard COPD therapy on this parameter have been largely neglected [15,17,18,19]. Several ex vivo studies have attempted to address this issue [15], but no comprehensive clinical analysis has been conducted yet. Furthermore, reported findings remain inconsistent, showing reduced, preserved, or even enhanced ciliary activity in smokers and COPD patients. These discrepancies likely reflect methodological differences as well as unaccounted pharmacological influences.

This issue has become increasingly relevant in the context of evolving COPD management strategies. The 2025 Global Initiative for Chronic Obstructive Lung Disease (GOLD) report introduces the revised GOLD ABE Assessment Tool (GOLD 2025 version), which replaces the former ABCD grouping. The new system emphasises exacerbation history over symptom severity, simplifying patient stratification into three categories [1].

- Group A: 0–1 moderate exacerbation in the past year and low symptom burden (Modified Medical Research Council—mMRC—Dyspnea Scale 0–1, COPD Assessment Test—CAT < 10);
- Group B: 0–1 moderate exacerbation but higher symptom burden (mMRC ≥ 2 and/or CAT ≥ 10);
- Group E: ≥2 moderate exacerbations or ≥1 leading to hospitalisation in the past year, regardless of symptom level.

Corresponding initial pharmacological recommendations are as follows:

- Group A: single long-acting bronchodilator—long-acting muscarinic antagonist (LAMA) or long-acting β_2_-agonist (LABA);
- Group B: dual bronchodilation with LAMA + LABA;
- Group E: LAMA + LABA as first choice, with LAMA + LABA + ICS recommended for patients with blood eosinophils ≥ 300 cells/μL.

This simplified overview reflects the growing emphasis on individualised therapy. Not all patients receive the same regimen, and many are treated with various combinations depending on their clinical profile. Consequently, it is important to reassess existing data through the lens of pharmacotherapy, considering both smoking-induced oxidative stress and the potential cilia-modulatory effects of inhaled and systemic medications in CBF assessment. Such a perspective may lead to more consistent interpretations of previously conflicting data.

Pathological changes in the nasal mucosa often mirror those in the lower airways, making upper airway sampling a valuable surrogate approach in respiratory research. However, drug-induced alterations in this region are mainly associated with systemic therapies such as oral corticosteroids, the phosphodiesterase (PDE)-4 inhibitor roflumilast, theophylline, or macrolide antibiotics. In contrast, inhaled bronchodilator combination therapy, the cornerstone of COPD management, acts predominantly on the bronchi. Consequently, measured CBF values may vary considerably between patients, reflecting differences in their therapeutic regimens.

Given these considerations, this article aimed to investigate the influence of prescribed COPD medications on MCC, focusing on ciliary motility under oxidative stress. The objective was to clarify their contribution to secondary complications and to identify potential strategies for optimising pharmacotherapy to enhance mucociliary function, prevent exacerbations, slow disease progression, and ultimately improve patients’ quality of life.

## 2. Airway Cilia

### 2.1. Airway Cilia Structure

In humans, microtubules form the main structural component of motile cilia. Cross-sectional electron microscopy images reveal their characteristic 9 + 2 arrangement, known as the axoneme, with nine peripheral microtubule doublets surrounding a central pair.

**Figure 1:**
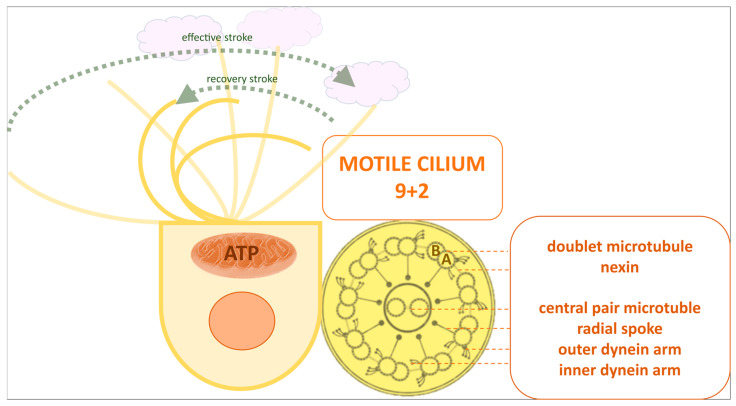
**Details of ciliary ultrastructure and kinematics.** The ciliary beat consists of two distinct phases: the effective stroke and the recovery stroke. In the effective stroke, the cilium extends fully and moves rapidly in one direction. During the recovery stroke, it bends and slowly retracts to its initial position, preparing for the next cycle. Note: ATP—adenosine triphosphate.

The A-B doublet microtubules are interconnected by the nexin-dynein regulatory complex. Outer and inner dynein arms, attached to the A microtubule, generate force for ciliary beating, with outer arms driving movement and inner arms modulating the size and shape of the ciliary bend. Radial spokes contribute to axonemal stability and regulate ciliary activity [20]. The adenosine triphosphate (ATP)-dependent dynein motor, together with structural proteins, stabilises adjacent microtubule doublets to restrict sliding and promote bending [21] (Figure 1).

Ciliary bending relies on cytoskeletal and regulatory dynein motor proteins. Additionally, ciliary motion is modulated by various phosphatases-protein phosphatase 1 (PP1) and protein phosphatase 2A, kinases-protein kinase A (PKA) and protein kinase C (PKC), and oxidant-generating systems such as nitric oxide synthases (NOS) and nicotinamide adenine dinucleotide phosphate (NADPH) oxidases (NOX/DUOX) [22].

### 2.2. Airway Cilia Kinematics

In humans, respiratory cilia beat at a slow constitutive rate owing to the spontaneous ATP-dependent activity of dynein. This basal, coordinated beating occurs at a frequency of approximately 9–16 Hz and represents a key kinematic parameter. CBF is modulated by a range of stimuli, including extracellular nucleotides such as ATP and uridine-5′-triphosphate, second messengers like cyclic adenosine monophosphate (cAMP), cyclic guanosine monophosphate (cGMP), and intracellular calcium ([Ca^2+^]i), as well as by pharmacological agents (e.g., β_2_-agonists), mechanical stress (e.g., viscous mucus), and environmental factors such as temperature, pH, airway hydration, electrolyte balance, and oxidative status [23,24,25,26,27,28,29].

This complex regulation is mediated by specialised membrane domains. The ciliary membrane, which is continuous with the apical plasma membrane, contains a repertoire of receptors and ion channels capable of detecting mechanical and chemical cues. These trigger intracellular signalling cascades that ultimately influence motility [30,31,32,33].

Among the principal intracellular regulators of CBF in airway epithelial cells are the second messengers cAMP, cGMP, and [Ca^2+^]i. These molecules integrate signals from diverse stimuli and play a pivotal role in the fine-tuning of ciliary activity [34,35,36,37,38,39,40,41].

Ciliary movement consists of an effective and a recovery stroke (Figure 1) within the airway surface liquid (ASL), a biphasic fluid layer that lines the airway epithelium. The lower layer, known as the periciliary layer (PCL), is a low-viscosity aqueous fluid surrounding the cilia, enabling their coordinated beating. The upper layer is a more viscous mucus gel that traps inhaled particles and pathogens, allowing their clearance via ciliary motion.

Mucus in the airways is secreted by both submucosal glands and goblet cells, with parasympathetic innervation in humans predominantly regulating submucosal gland secretion via M3 muscarinic receptors. The biochemical composition of mucus undergoes pathological changes that contribute to COPD progression. Its rheological properties are determined primarily by water and mucin content, primarily mucin 5B (MUC5B) and mucin 5AC (MUC5AC), the latter of which is markedly overproduced in COPD. MUC5AC is predominantly produced by goblet cells within the tracheobronchial epithelium. In contrast, MUC5B is mainly secreted by submucosal glands and their ducts, with only minor contributions from goblet cells in the distal airways.

Hydration of the ASL, particularly the PCL, is maintained by serous cells located in submucosal glands. These cells actively secrete chloride and bicarbonate, generating an osmotic gradient that drives water movement into the lumen. During airway inflammation, this balance is disrupted. Serous cells may transdifferentiate into mucus-producing cells, leading to reduced fluid secretion and impaired MCC [42,43,44,45,46,47,48,49].

Fluid secretion in the airways is governed by the cystic fibrosis transmembrane conductance regulator (CFTR), the epithelial sodium channel (ENaC), and Ca^2+^-activated chloride channels (CaCCs), such as transmembrane member 16A (TMEM16A), which respond to Ca^2+^ mobilisation via acetylcholine agonists and ATP. CFTR, a cAMP-activated Cl^−^ channel, also responds directly to calmodulin-mediated Ca^2+^ signalling and interacts with Ca^2+^ pathways to regulate secretion [50,51,52,53,54,55].

Importantly, ATP, which is vital for both fluid transport and ciliary function, is primarily synthesised during cellular respiration in mitochondria. Due to their size, mitochondria are excluded from cilia and instead localise near ciliated cells, where they provide energy for ciliary beating and protect respiratory epithelial cilia from oxidative damage through mitochondrial uncoupling proteins, albeit at the cost of reduced mitochondrial efficiency [56].

## 3. Reactive Oxygen Species and Oxidative Stress

Oxidative damage in tissues is caused by reactive oxygen species (ROS) produced in various cellular compartments, including the cytoplasm, cell membrane, endoplasmic reticulum (ER), mitochondria, and peroxisomes, as part of cellular aerobic metabolism. Mitochondria are the primary contributors to ROS production, accounting for approximately 90% of all cellular ROS. These highly reactive molecules are also generated by a variety of enzymes, such as NADPH oxidases, xanthine oxidase, NOS, and peroxisomes. Additionally, ROS can be produced secondarily in response to external stimuli, including ionising and ultraviolet (UV) radiation, drugs, tobacco, and environmental pollutants. The folding of proteins and the formation of disulphide bonds in the ER also have the potential to release oxidants.

ROS are capable of accepting electrons, generating unstable molecules such as superoxide anions (O_2_^•−^), hydrogen peroxide (H_2_O_2_), hydroxyl radical (^•^OH), and singlet oxygen (^1^O_2_), which are generated by various cell types. Within the inner mitochondrial membrane, the primary objective of the electron transport chain is the reduction of oxygen molecules to produce water (H_2_O).

Due to their charge, O_2_^•−^ cannot cross biological membranes. They are unstable, highly reactive, and the most abundant ROS in mitochondria. O_2_^•−^, are rapidly converted into H_2_O_2_ and ^•^OH [57,58,59,60].

### 3.1. Intracellular Redox Systems of Motile Cilia

Motile cilia contain both oxidant-generating and antioxidant systems, which play a crucial role in their function.

**Figure 2:**
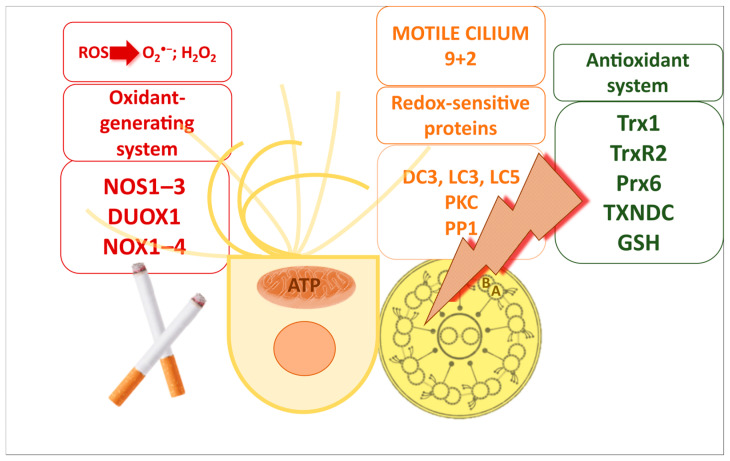
**Redox homeostasis in the motile cilium of airway epithelial cells.** Note: ROS—reactive oxygen species; O_2_^•−^—superoxide anions; H_2_O_2_—hydrogen peroxide; NOS1–3—nitric oxide synthase 1–3; DUOX1—dual oxidase 1; NOX1–4—NADPH oxidase 1–4; Trx1—thioredoxin 1, TrxR2—thioredoxin reductase 2, Prx6—peroxiredoxin 6; TXNDC—thioredoxin domain-containing proteins; GSH—glutathione system; DC3, LC3, and LC5—outer dynein arm subunits; PKC—protein kinase C; PP1—protein phosphatase 1; ATP—adenosine triphosphate.

The oxidant-generating system comprises three subtypes of NOS1–3, NADPH oxidase membrane-bound enzymes (DUOX1, NOX1–4), and mitochondria (Figure 2). These components exhibit specific localisation: NOS1 and DUOX1 are situated along the ciliary membrane, NOX1–4 are found on the apical surface of the cell membrane, NOS2 is located in the cytoplasm, and NOS3 is associated with the basal body. Together with mitochondria, which are located near the basal bodies, these components produce O_2_^•−^ and H_2_O_2_.

The antioxidant system consists of the thioredoxin (Trx) and glutathione (GSH) systems. Crosstalk between these systems arises from their functional overlap, as both serve as hydrogen donors for numerous metabolic enzymes. In relation to airway cilia, the antioxidant system is primarily located within the ciliary matrix and the cytoplasm of airway ciliated cells. Motile cilia, in particular, are rich in thiol-dense and thiol-regulatory proteins, including Trx1, thioredoxin reductase 2, and peroxiredoxin 6. Furthermore, several thioredoxin domain-containing (TXNDC) proteins have been identified along the axoneme, in close proximity to dynein (Figure 2) [22].

In contrast, the GSH-glutaredoxin system has not been identified in cilia. There is only indirect evidence suggesting that GSH precursors prevent the loss of motile cilia, restore ciliary beating, and reduce mucus viscosity in normal human bronchial epithelial cell cultures [61]. Additional evidence points to a negative role of glutathione transferase (GST) theta, which is abundant in the axoneme of lateral motile cilia in sea urchin embryos, in the regulation of ciliary motility [62]. This aligns with recent findings by Koenitzer et al. [63], which demonstrate increased expression of GST (GSTA1, GSTA2) and nuclear factor erythroid 2-related factor 2 target genes under conditions of elevated ROS levels.

Cellular redox systems play numerous roles, including defence against oxidative stress and the regulation of cell growth versus cell death [64].

In mitochondria, physiological levels of O_2_^•−^ and H_2_O_2_ participate in redox signalling and influence the balance between ROS production and the protective activity of the antioxidant defence system. During oxidative stress, their production is markedly elevated. The mitochondrial antioxidant system comprises superoxide dismutases (SOD), which convert O_2_ to H_2_O_2_. Zn-Cu SOD (SOD1) is found in the cytosol and mitochondrial intermembrane space, while Mn-SOD (SOD2) is located in the mitochondrial matrix. Additionally, catalase, glutathione peroxidases (GPX), and TrxR further break down H_2_O_2_ into H_2_O and O_2_ [56].

### 3.2. Impact of Oxidative Stress on Airway Cilia

#### 3.2.1. Ciliation of Epithelial Cells

In animal culture models, oxidative stress increases epithelial GSH levels, induces DNA damage, and promotes lipid peroxidation. It causes a time-dependent decrease in epithelial cilia coverage, which is partially mitigated by supplementation with vitamins E and C, either individually or in combination [65]. Loss of cilia has also been documented in human bronchial cultures exposed to hyperoxia [66] and in patients requiring intubation, where it was associated with impaired mucociliary transport [67].

The reduction in epithelial ciliation is primarily attributed to intracellular ROS accumulation, cellular energy depletion, and a decreased availability of antioxidant enzymes [56,68,69,70]. Ultrastructural abnormalities linked to ciliary dysfunction have also been reported in patients with chronic airway inflammation. These changes progress with disease severity and with the intensity of causal factors, ultimately leading to a marked reduction in the number of ciliated cells and a parallel increase in cells with complete ciliary loss [71,72,73,74]. This loss of airway cilia, characterised by axonemal disintegration and misorientation of the remaining basal bodies, contributes to impaired MCC, as shown in an in vitro model of SARS-CoV-2 infection in human bronchial epithelium [75].

Oxidative stress induces a marked reduction in the number of ciliated epithelial cells, diminishing overall ciliation and impairing MCC, thereby increasing susceptibility to airway infection.

#### 3.2.2. Cilia Length

Oxidative stress can significantly alter the length of motile cilia in the respiratory tract. Excessive ROS production damages ciliary structures, disrupts axonemal integrity, and interferes with ciliary assembly and repair, ultimately leading to shortened cilia [70,76,77]. Models of mucociliary function indicate that effective MCC requires cilia to exceed the 6–7 μm depth of the airway surface fluid in order to generate sufficient force within the mucus layer [38,78].

Oxidative stress-mediated shortening of motile cilia compromises their ability to generate effective force within the mucus layer, reducing MCC and heightening vulnerability to respiratory pathogens.

#### 3.2.3. Ciliary Orientation

Although alterations in ciliary orientation are most commonly described in PCD [79], similar changes have also been demonstrated in a human in vivo study, where infection-induced inflammation of the upper respiratory tract resulted in ciliary disorientation. This effect was likely mediated by oxidative stress and was strongly correlated with delayed MCC [80].

These observations indicate that oxidative stress–driven ciliary disorientation, although initially described in infection-related inflammation, may also be relevant in chronic airway diseases such as COPD, where persistent oxidative burden contributes to impaired MCC.

#### 3.2.4. Ciliary Cellular Components

Oxidative stress can damage cellular biomolecules, including lipids, proteins, and DNA. H_2_O_2_, a O_2_^•−^ product that is permeable to biological membranes, can undergo the Fenton reaction in the mitochondrial matrix, producing the highly reactive ^•^OH. Excessive ROS production leads to increased mitochondrial DNA damage, as it can disrupt antioxidant defence mechanisms in the cells [58,65].

Evidence suggests that epithelial cells with a higher proportion of ciliated cells exhibit lower ROS levels, likely due to elevated expression of mitochondrial uncoupling proteins, compared to goblet or secretory cells. However, these proteins reduce the efficiency of mitochondrial respiration in producing ATP. In contrast, epithelia with fewer ciliated cells rely more heavily on mitochondrial respiration for ATP generation. These findings indicate that ciliated cells prioritise mitochondrial protection over ATP production [56].

Oxidative stress also negatively impacts cellular phospholipids, particularly phosphatidylinositol 4,5-bisphosphate (PI(4,5)P_2_) [39], which, along with phosphatidylinositol 4-phosphate (PI(4)P), are the most extensively studied ciliary phosphoinositides. PI(4)P is the predominant phosphoinositide in the ciliary membrane, whereas PI(4,5)P2 is primarily confined to the proximal region of the cilium and the ciliary base.

This unique distribution of PI(4)P in the ciliary membrane is maintained by ciliary phosphatidylinositol 5-phosphate (PI(5)P). Loss of this enzyme disrupts the PI(4)P balance, resulting in its replacement by PI(4,5)P_2_, which regulates Hedgehog signalling and contributes to slower ciliary beating in motile cilia [40,41]. Additionally, PI(4)P plays a crucial role in the formation of primary cilia [81].

Dynein ATPases, which power ciliary movement, are highly sensitive to alterations in the redox environment within each cilium. Their activity is tightly regulated by phosphoregulatory enzymes, including PKA, PKC, and PP1, which themselves are subject to reversible or irreversible redox modifications [22]. In addition to these kinases and phosphatases, motile respiratory cilia are enriched in redox-regulatory proteins such as Trx and TXNDC proteins. Electron microscopy studies have identified Trx-like protein 2 in association with the microtubular structures of airway cilia [82,83], highlighting its protective role. Trx-mediated redox regulation involves key dynein components, particularly outer dynein arm subunits such as DC3, LC3, and LC5, thereby stabilising axonemal function under oxidative conditions (Figure 2). This intricate regulatory network safeguards ciliary motility against oxidative stress and ensures the preservation of effective MCC.

Beyond the direct effects of enzymatic and regulatory protein modifications, cigarette smoke exacerbates ciliary dysfunction by promoting the degradation of other essential ciliary proteins. Recent studies have demonstrated that cigarette smoke triggers degradation of ciliary proteins, such as intraflagellar transport proteins, via autophagy, with histone deacetylase 6 playing a crucial role. This mechanism directly impairs normal ciliary growth and function in the context of COPD [84].

Beyond oxidative stress, structural defects of the cilia have been reported in COPD patients, representing an additional factor that severely compromises overall ciliary performance [85].

In summary, oxidative stress disrupts mitochondrial integrity, phosphoinositide balance, and dynein regulation, all of which are critical for maintaining effective ciliary function. Understanding these redox-sensitive mechanisms may offer novel therapeutic avenues for preserving MCC in chronic airway diseases.

#### 3.2.5. Ciliary Kinetics

Collectively, changes in the number of ciliated cells, cilia count, cilia length, and alterations in individual structural components influence ciliary movement, potentially reducing MCC efficiency [74,86,87]. Mitigating the causative factors, such as tobacco smoking, or enhancing antioxidant defences, may help slow down or partially reverse damage to these motile organelles. Early intervention to counteract the negative effects on respiratory defence mechanisms is critical for limiting disease progression and preventing irreversible remodelling changes.

Studies of antioxidant expression in motile epithelial cells have demonstrated higher mRNA levels of cytosolic antioxidants, including SOD1, catalase, and GPX4, compared to goblet and secretory cells. Conversely, mitochondrial antioxidants such as SOD2 and GPX2 were observed at lower levels in these cells. In this context, SOD catalyses the conversion of O_2_^•−^ into H_2_O_2_, catalase transforms H_2_O_2_ into H_2_O and O_2_, and GPX reduces H_2_O_2_ to H_2_O while also converting lipid hydroperoxides into alcohols. These data indicate that antioxidants play a crucial role in protecting motile epithelial cells from ROS-induced damage, thereby supporting effective mucociliary transport [56].

Antioxidant mechanisms in motile epithelial cells are essential for preserving ciliary kinetics and MCC efficiency, highlighting the importance of early mitigation of oxidative stress to maintain airway defence.

### 3.3. Impact of Oxidative Stress on Airway Mucus Secretion and Composition

#### 3.3.1. Composition and Function of Respiratory Mucus

Respiratory mucus is primarily composed of the gel-forming mucins MUC5B and MUC5AC, which are essential for airway clearance, airway protection, and immune homeostasis in the lungs [88,89]. The balance between these mucins determines mucus rheology and its ability to efficiently transport inhaled particles and pathogens.

#### 3.3.2. Effects of Cigarette Smoke and Inflammation on Mucin Production

Exposure to cigarette smoke and recruitment of neutrophils during inflammation elevate the burden of proteases and oxidants in the lungs. These factors activate intracellular signalling pathways, particularly those involving epidermal growth factor, leading to upregulation of MUC gene expression and subsequent mucin overproduction. This cascade causes mucous metaplasia, characterised by goblet cell hyperplasia, submucosal gland hypertrophy, and enhanced mucin secretion mediated via myristoylated alanine-rich C kinase substrate [90]. In COPD, airway secretions show a relative increase in MUC5B over MUC5AC compared with asthma patients [91].

#### 3.3.3. Neurogenic Regulation and Protease Effects

In response to cigarette smoke in COPD, inflammatory cells release proteolytic enzymes that degrade vasoactive intestinal peptide and neutral endopeptidase, removing inhibitory modulation of neurogenic mucus secretion. The resulting unregulated neuropeptide signalling increases tachykininergic mucus output, further contributing to hypersecretion [92].

#### 3.3.4. Structural Changes in Goblet Cells and Submucosal Glands

Under normal conditions, submucosal glands predominate in cartilaginous airways, with a typical gland-to-goblet cell ratio of 40:1. In hypersecretory conditions, goblet cell hyperplasia and metaplasia affect distal airways, leading to excessive mucus production that is difficult to clear via MCC or coughing [92]. Such changes are strongly linked to morbidity and mortality in COPD [93].

#### 3.3.5. Biophysical Properties of Mucus and Mucociliary Clearance Efficiency

The effectiveness of MCC is determined by mucus gel elasticity, viscosity, and the sol layer depth. Cigarette smoke impairs these properties by inducing airway dehydration and increasing mucus viscosity. Intracellular cAMP ([cAMP]i) and [Ca^2+^]i regulate electrolyte and macromolecular secretion, with basal secretion relying on their interplay, and transient secretion mainly driven by cholinergic elevation of [Ca^2+^]i [94].

Oxidative stress and inflammatory insults, particularly from cigarette smoke, drive goblet cell hyperplasia and mucin overproduction, altering mucus composition and viscosity. Together with ciliary shortening and loss, these changes impair MCC and contribute to airway obstruction in chronic respiratory diseases.

### 3.4. Oxidative Stress in COPD

#### 3.4.1. Sources of Reactive Oxygen Species in the Lungs

The lungs are constantly exposed to ROS, with mitochondria serving as the primary endogenous source through superoxide radical production during cellular respiration. These radicals can transform into the highly reactive ^•^OH, which is elevated in patients with COPD, or react with nitric oxide to form peroxynitrites. ROS also arise as part of the body’s response to bacterial and viral infections.

External sources of ROS include environmental oxidative gases, UV radiation, and nanoparticles from industrial pollution and vehicle exhaust. Among these, cigarette smoke remains the most significant etiological factor driving COPD. Importantly, oxidative stress persists even after long-term smoking cessation, contributing to ongoing disease progression, likely through sustained mitochondrial activity [95].

#### 3.4.2. Antioxidant Defence Mechanisms

To counteract oxidative stress, the lungs possess an antioxidant defence system, with reduced GSH as its principal component, approximately 20% of which is mitochondrial. Other antioxidants are found in the airway surface fluid, including ascorbic acid, α-tocopherol, and uric acid, while sulfhydryl groups on albumin and mucin surfaces contribute to local epithelial protection [96].

#### 3.4.3. Pathophysiological Consequences of Oxidative Stress

ROS mediate tissue damage through mechanisms such as lipid peroxidation, leading to impaired phagocyte function, muscle dysfunction, tissue remodelling, cell growth or death, DNA damage, bronchoconstriction, mucus hypersecretion, stimulation of pro-inflammatory signalling, antibody production, and mitochondrial injury. Damaged mitochondria in turn become a secondary ROS source, perpetuating a vicious cycle that drives COPD progression [97]. Persistent oxidative stress not only exacerbates inflammation but may also underlie treatment resistance in certain patients.

#### 3.4.4. Therapeutic Implications

Current COPD management predominantly targets bronchodilation to relieve hyperinflation and enhance lung mechanics. However, a significant subset of patients exhibits a limited or poor response to standard therapeutic regimens. For these individuals, adjunctive anti-inflammatory strategies, such as administration of corticosteroids (CSs) and PDE inhibitors, have been considered [1,14]. At this stage, patients frequently present with pronounced symptoms such as dyspnoea, mucus overproduction, and a worsening cough, all of which reflect underlying impaired ciliary function. However, GOLD 2025 [1] guidelines offer minimal therapeutic direction for enhancing MCC, apart from mucolytics, despite the known cilia-stimulatory effects of LABAs [98,99].

#### 3.4.5. Ciliary Dysfunction in COPD

Research on ciliary kinetics in COPD patients consistently demonstrates hallmark features of dysfunction, including reduced CBF, shortening of motile cilia, ciliary loss, and dyskinesia [15,71,76,85,87,100,101,102,103]. These alterations, partly driven by cigarette smoke–induced degradation of ciliary proteins as discussed previously, are further exacerbated by oxidative stress from depleted antioxidants and inadequate dietary intake [84,104]. The resulting disruption of cellular homeostasis directly impacts critical signalling pathways, particularly those regulating Ca^2+^, linking ciliary dysfunction to impaired mucus clearance and altered airway responses.

## 4. Calcium Signalling

The balance of Ca^2+^ between extracellular and intracellular spaces is maintained through the coordinated interaction of the ER, mitochondria, nucleus, plasma membrane, ion channels, and regulatory proteins. Ca^2+^ enters the cell through cation channels in the plasma membrane and is released from intracellular stores in a tightly regulated manner to sustain physiological levels essential for cellular function. These finely tuned processes are particularly vulnerable to disruption in chronic respiratory diseases such as COPD [105].

Ca^2+^ is transported from the extracellular space or cytosol into the ER or mitochondria through store-operated Ca^2+^ entry channels and voltage-dependent anion channels. The key effectors involved in this process are stromal interaction molecule (STIM) 1 and the mitochondrial Ca^2+^ uniporter. At mitochondria-associated membranes, the ER releases Ca^2+^ via inositol trisphosphate receptors (IP_3_Rs), enabling tight coupling between the two organelles. This intricate interplay between Ca^2+^ and mitochondria is crucial for regulating mitochondrial function and dynamics [106].

Excessive cytoplasmic Ca^2+^ can trigger irreversible cellular changes leading to cell death. To prevent this, cells rely on specialised mechanisms that maintain Ca^2+^ at safe, low levels. These regulatory systems operate both at the plasma membrane and at the ER membrane.

At the plasma membrane, the main mechanisms are the plasma membrane Ca^2+^-ATPase (PMCA) and the Na^+^/Ca^2+^ exchanger, which remove excess Ca^2+^ from the cytoplasm. At the ER membrane, the sarco/endoplasmic reticulum Ca^2+^-ATPase (SERCA) actively pumps Ca^2+^ into the ER. Both PMCA and SERCA belong to the P-type ATPase family, using ATP to drive Ca^2+^ transport against its concentration gradient.

When ER Ca^2+^ levels decline, the ER-resident sensors STIM1 and STIM2 translocate to ER–plasma membrane junctions and activate store-operated Ca^2+^ channels (SOCCs), which are formed by Orai proteins (Orai_1-3_). This allows extracellular Ca^2+^ to enter the cytoplasm, replenishing ER stores and stimulating Ca^2+^ pumps to restore homeostasis. Dysregulation of these pathways is associated with various diseases, including COPD [107].

### 4.1. Calcium Signalling in Patients with COPD

Airway Ca^2+^ signalling regulates a range of physiological processes, such as smooth muscle contraction, neuronal excitability, mucus secretion, ciliary movement, cell migration, cytokine release, and epithelial repair [108,109,110,111,112,113,114]. It is pivotal in the pathophysiology of COPD, where ATP-producing mitochondria exhibit abnormal accumulation of [Ca^2+^]i. This imbalance promotes local ROS production, further driving the activation of apoptotic pathways [105,115]. Despite the widely accepted fact that oxidative stress significantly contributes to COPD progression, oxidative stress persists even after cigarette smoking cessation. Therefore, research into mitochondria as a source of ROS has gained increasing importance in understanding the pathobiology of COPD [116].

Disruption of Ca^2+^ signalling in COPD has also been documented at multiple levels, including impaired ER Ca^2+^ release and reduced Ca^2+^ influx, although baseline cytosolic Ca^2+^ often remains comparable to that of healthy individuals. Nevertheless, acute cigarette smoke exposure can significantly reduce cytosolic Ca^2+^, further disturbing Ca^2+^ homeostasis.

Notably, research has identified 55 genes involved in Ca^2+^ signalling, with Orai_3_ being the only gene significantly affected in smokers, irrespective of COPD status. These findings highlight impaired Ca^2+^ signalling in the airway epithelium of smokers, with Orai_3_ playing a central role in this dysfunction, particularly in controlling ciliary motion [117,118].

The modulation of Ca^2+^ signalling pathways is not only relevant to the cellular impact of nicotine but also plays a pivotal role in the mechanisms of action of LAMAs and LABAs, both of which are cornerstone bronchodilators in the management of COPD.

This prompts the question of whether smoking-induced alterations in Ca^2+^ signalling could indirectly influence the pharmacodynamics of bronchodilator therapy.

To understand this interaction further, it is important to consider that at least three subtypes of muscarinic receptors (M_1_, M_2_, and M_3_) are expressed in the lungs of mice, pigs, and humans [119,120].

### 4.2. The Role of Calcium in Ciliary Kinetics

CBF is tightly regulated by intracellular Ca^2+^, primarily through the Ca^2+^–calmodulin complex, which interacts with cyclic nucleotides cAMP and cGMP to modulate ciliary motion. Ca^2+^ is mobilised from intracellular stores via IP_3_ signalling or enters through membrane ion channels following activation of specific receptors, such as purinergic P_2_Y_2_ or muscarinic M_1_/M_3_. CBF responses to second messengers are typically biphasic: an initial Ca^2+^–calmodulin- and PKG-dependent phase, followed by a sustained phase maintained by PKA activity, which can operate independently of Ca^2+^. Disruption of the nitric oxide (NO)–cGMP–PKG pathway can abolish Ca^2+^-mediated ciliary stimulation, highlighting the importance of coordinated cross-talk between Ca^2+^ and cyclic nucleotide signalling for effective ciliary function [24,121,122,123,124,125].

### 4.3. The Role of Calcium in Muscarinic Receptor–Antagonist Signalling

**Figure 3:**
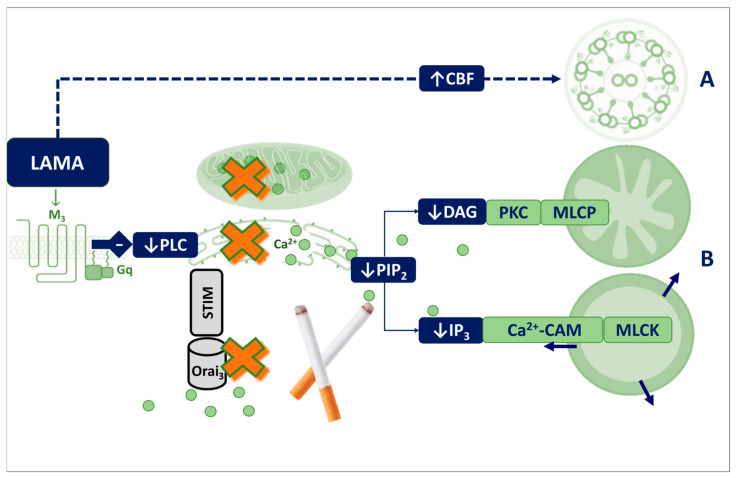
**LAMA-mediated cilia-stimulatory and bronchodilatory effects in COPD.** LAMA-induced bronchodilation (B) occurs through blockade of M_3_ muscarinic receptors (M_3_MRs) in airway smooth muscle (blue), resulting in reduced intracellular Ca^2+^ signalling via PLC inhibition. In parallel, LAMAs exert a cilia-stimulatory effect (blue dashed line) (A) and selectively suppress pathological MUC5AC secretion while preserving protective MUC5B. In COPD, Ca^2+^ signalling is dysregulated at multiple levels: diminished Ca^2+^ influx through the Orai3 ion channel, depleted ER Ca^2+^ stores, and mitochondrial Ca^2+^ overload (orange cross), together impairing epithelial defence and mucus clearance. Note: LAMA—long-acting antimuscarinic drugs; CBF—ciliary beat frequency; PLC—phospholipase C; PIP_2_—phosphatidylinositol 4,5-bisphosphate; IP_3_—inositol 1,4,5-trisphosphate; Ca^2+^-CAM—calcium-calmodulin complex; MLCK—myosin light-chain kinase; DAG—diacylglycerol; PKC—protein kinase C; MLCP—myosin light-chain phosphatase; Orai_3_—calcium release-activated calcium modulator 3; STIM—stromal-interacting molecule [13,85,115,116,118,126,127,128].

Inhaled short-acting muscarinic receptor antagonists (SAMAs) and LAMAs exert their bronchodilator effects by blocking Gq-protein-coupled M_3_ receptors. This inhibition lowers [Ca^2+^]i levels in the airways, preventing phospholipase C (PLC) β-mediated hydrolysis of (PI(4,5)P_2_) and the subsequent formation of diacylglycerol and inositol triphosphate (IP_3_), ultimately promoting airway smooth muscle (ASM) relaxation [126,127,128] (Figure 3).

Muscarinic receptors are also involved in regulating ciliary beating [129,130]. Among the subtypes, M_1_, M_2_, and M_3_ play a role in cilia-driven particle transport: M_3_ receptors enhance transport, M_2_ receptors suppress it, and M_1_ can compensate when M_2_ and M_3_ are absent. Selectively targeting M_3_ receptors while sparing M_1_ may preserve the bronchodilator benefits of anticholinergics while minimising potential effects on ciliary function [119,120].

Contrary to earlier beliefs, recent research has demonstrated that LAMA-mediated cilia stimulation appears to occur independently of classical second messengers, including Ca^2+^, cAMP, PKA, and purinergic signalling (Figure 3), cAMP, PKA, and purinergic signalling [13]. This implies that LAMA-induced ciliary stimulation is largely unaffected by COPD-related Ca^2+^ disturbances, whereas LABAs might require additional support due to PDE-driven cAMP depletion [131,132].

A few experiments have investigated the effects of anticholinergics on ciliary movement. Existing studies provide insufficient evidence of negative effects and primarily focus on SAMAs, such as ipratropium bromide, or non-selective long-acting tertiary amines like atropine [133,134]. At the time, classical LAMA (tiotropium) and newer agents (glycopyrronium, umeclidinium, aclidinium, and revefenacin) had not yet been developed or widely introduced into clinical practice. While some research suggests that anticholinergics may stimulate ciliary movement, the proposed mechanism typically involves muscarinic receptors [129,130,135]. This historical context highlights the limited evidence from older studies and underscores the need to investigate the effects of newer LAMAs on ciliary function in COPD.

Ca^2+^ signalling indirectly modulates CFTR function by amplifying its response to muscarinic agonists, enhancing Cl^−^ secretion and reducing Na^+^ reabsorption via ENaC. This maintains ASL hydration and supports effective MCC [136,137]. LAMA-mediated inhibition of M3 receptors reduces cytoplasmic Ca^2+^, which can limit MUC5B and MUC5AC secretion, potentially normalising mucus overproduction but also altering ASL properties [138]. The overall impact on MCC remains complex, as reduced hydration and protective mucus may create conditions favourable for bacterial growth [49].

Recent studies emphasise mucin-specific therapeutic strategies that selectively reduce pathological mucus production. In particular, targeting MUC5AC is of clinical relevance, as its overproduction has been implicated in severe muco-obstruction in COPD, whereas a marked suppression or loss of MUC5B secretion may impair host defence. In this context, tiotropium has been shown to selectively inhibit MUC5AC production in virally infected epithelial cells without compromising MCC, thereby reducing pathological mucus while preserving essential protective mechanisms of the airways [139,140,141].

Nicotine-induced changes also affect bronchodilator targets: cigarette smoke triggers CFTR internalisation, lysosomal Ca^2+^ release, and increased Na^+^ absorption, contributing to ASL dehydration and mucus stasis [142]. TMEM16A (CaCCs) expression remains largely unchanged, which may protect some ion channel function during LAMA therapy [143].

Viscous mucus on the ciliary surface mechanically stimulates ATP release, but COPD-related Ca^2+^ signalling impairments [117,118] can reduce ATP-triggered Ca^2+^ influx. Transient receptor potential cation channel subfamily V member 4 (TRPV4) mediated Ca^2+^ entry may help compensate for these deficits [144], highlighting the complex interplay between Ca^2+^ signalling, mucus properties, and ciliary function in COPD.

Together, these findings emphasise that LAMAs not only provide bronchodilation through ASM relaxation but also directly preserve ciliary function and selectively suppress pathological MUC5AC secretion without diminishing protective mucus. Importantly, LAMA-mediated cilia stimulation appears to occur independently of Ca^2+^ signalling. This dual effect supports airway mechanics and epithelial homeostasis in COPD, highlighting LAMAs as key modulators of both airway function and mucus regulation.

### 4.4. The Role of Calcium in β_2_-Agonists Signalling

Gs protein-coupled β_2_-adrenergic receptors are abundant in ASM, which, unlike submucosal glands and blood vessels, lacks direct sympathetic innervation. Instead, ASM responds to circulating catecholamines in the bloodstream. Stimulation of β_2_-adrenergic receptors by agonists triggers adenylyl cyclase, generating cAMP. This cascade promotes ASM relaxation by enhancing sequestration of [Ca^2+^]i.

β_2_-receptor agonists are generally considered to reduce Ca^2+^ influx into ASM cells. Supporting this mechanism, several studies have shown that IP_3_Rs are phosphorylated by cAMP-dependent PKA, thereby diminishing Ca^2+^ release from the ER in response to IP_3_. Interestingly, isoprenaline induces spatially distinct alterations in cytosolic Ca^2+^ within ASM: it raises Ca^2+^ levels in the peripheral cytosol through an extracellular Ca^2+^-dependent process, which is inhibited by ryanodine, while simultaneously lowering Ca^2+^ concentration in the central cytosol of the same cell [145].

PKA consists of two regulatory and two catalytic subunits. Rising intracellular cAMP binds to the regulatory subunits, liberating catalytically active PKA. In ASM, these active subunits phosphorylate multiple targets, including the transcription factor cAMP response element-binding protein, PLC, IP_3_R, myosin light-chain kinase, large-conductance voltage Ca^2+^-activated potassium ion channels, and the β_2_-adrenergic receptor itself [146]. Collectively, these interactions culminate in ASM relaxation.

However, the mechanisms underlying ASM relaxation extend beyond PKA [147,148]. cAMP also stimulates the exchange proteins directly activated by cAMP (Epac1 and Epac2). Epac promotes the exchange of guanosine-5′-diphosphate for guanosine-5′-triphosphate (GTP) on G-proteins, which attenuates Rho signalling and contributes to ASM relaxation independently of PKA [149,150].

**Figure 4:**
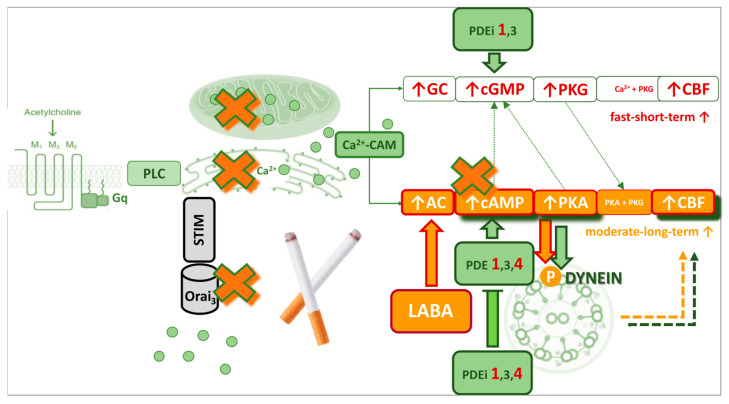
**Interplay of LABA and Phosphodiesterase Signalling Pathways in COPD Airways with Emphasis on Ciliary Regulation.** LABA-mediated cilia stimulation (orange dashed line) occurs through activation of adenylyl cyclase (AC), increasing intracellular cAMP and activating PKA. This leads to phosphorylation of the outer dynein arm light chain (orange) following β2-adrenergic receptor (β2AR) activation on ciliated cells. In COPD, Ca2+ signalling is dysregulated at multiple levels: diminished Ca2+ influx through the Orai3 ion channel, depleted ER Ca2+ stores, and mitochondrial Ca2+ overload (orange cross). The latter triggers compensatory activation of uncoupling proteins (UCP2, UCP5), which reduce ROS generation but also impair oxidative phosphorylation, leading to reduced ATP availability for dynein motors and compromised ciliary beating. In parallel, increased PDE activity reduces intracellular cAMP (orange cross), impairing both anti-inflammatory signalling and airway smooth muscle relaxation. PDE1A, a Ca2+-dependent enzyme localised within the ciliary axoneme, fine-tunes cAMP–driven regulation of CBF. In COPD, conflicting mechanisms exist: while PDE1A expression is reduced in bronchial epithelium, oxidative stress enhances PDE1A activity, leading to net cAMP degradation. PDE4 is also upregulated, lowering cAMP further; PDE4 inhibitors restore CBF (green dashed line) but only in the presence of LABA. PDE3 is pharmacologically relevant mainly in the context of dual PDE3/4 inhibitors (e.g., ensifentrine), where PDE3 inhibition contributes to bronchodilation, while PDE4 inhibition supports anti-inflammatory effects and ciliary stimulation (green dashed line). Together, impaired Ca2+ homeostasis, mitochondrial bioenergetic compromise, and PDE-driven cAMP degradation converge to disrupt ciliary motility and MCC in COPD. Note: LABA—long-acting β2-agonists; CBF—ciliary beat frequency; PLC—phospholipase C; Ca2+-CAM—calcium-calmodulin complex; Orai3—calcium release-activated calcium modulator 3; STIM—stromal-interacting molecule; GC—guanylate cyclase; cGMP—cyclic guanosine monophosphate; PKG—cyclic GMP-dependent protein kinase G; AC—adenylyl cyclase; cAMP—cyclic adenosine monophosphate; PKA—cyclic AMP-dependent protein kinase A; PDEi—phosphodiesterase inhibitors (PDEi 1, 3, 4); PDE—phosphodiesterase (PDE 1, 3) [12,16,85,115,118,132,151,152,153,154,155,156,157,158,159].

The role of cAMP is tightly regulated by PDEs, of which 11 families have been identified. PDE4 is the most abundant in ASM, whereas PDE1A is crucial for MCC. PDE1A expression is found both in ciliated cell bodies and within the cilia themselves, where it modulates CBF in response to [Ca^2+^]i (Figure 4) [151].

The cAMP and Ca^2+^ signalling pathways have traditionally been viewed as largely separate. However, emerging evidence reveals connections between cAMP and ion channels that mediate Ca^2+^ entry into cells, particularly SOCCs, which restore ER Ca^2+^ stores and link Ca^2+^ homeostasis with cAMP signalling, including local Ca^2+^–cAMP cross-talk mediated by Ca^2+^-dependent PDE1A that fine-tunes CBF in ciliated cells [151,160].

Beyond Ca^2+^ regulation, cAMP shapes multiple processes relevant to ASM and epithelial function, including the production of inflammatory mediators and extracellular matrix, cell proliferation and migration, mucus secretion, wound repair, and the control of ciliary motion (Figure 4). Moreover, LABA can partially counteract LAMA-mediated surface dehydration through cAMP-dependent ENaC-mediated Na^+^ secretion [161]. However, this effect may be minimal, as tobacco smoke enhances ENaC activity, further reducing ASL height [142].

Taken together, β_2_-agonists promote ASM relaxation primarily through cAMP-dependent modulation of Ca^2+^ dynamics, complemented by Epac-mediated suppression of Rho signalling and local Ca^2+^–cAMP cross-talk via PDE1A in ciliated epithelial cells. This integrated regulation of smooth muscle tone, mucus hydration, and ciliary activity underscores the central therapeutic value of β_2_-agonists in COPD, while highlighting the interplay between Ca^2+^ signalling, PDE activity, and ciliary function.

### 4.5. The Role of Calcium in Corticosteroid Signalling

Interleukin (IL)-13 is a central mediator linking type 2 inflammation with mucus hypersecretion, airway remodelling, and ciliary dysfunction. It promotes goblet cell metaplasia, increases MUC5AC production, and reduces CBF, impairing MCC [162,163]. Mechanistically, IL-13 modulates Ca^2+^ signalling in both ASM and epithelial cells: it amplifies Ca^2+^ release from intracellular stores in ASM, enhancing contractile responses to bronchoconstrictors such as histamine, and disrupts mitochondrial Ca^2+^ homeostasis in epithelial cells, promoting oxidative stress and apoptosis. These dual effects contribute to airway narrowing and epithelial barrier dysfunction, highlighting IL-13 as a mechanistic link between inflammation and Ca^2+^ dysregulation.

Inhaled CSs (ICSs), including fluticasone, budesonide, and beclomethasone, attenuate IL-13-driven effects by reducing Th_2_ cytokine activity, limiting goblet cell metaplasia, restoring MUC5B secretion, and indirectly supporting Ca^2+^-dependent ciliary function, thereby preserving MCC [164,165]. However, ICSs are less effective in patients with neutrophilic inflammation or corticosteroid resistance. Clinical data suggest that ICS do not significantly impair MCC in COPD patients, despite their effects on Ca^2+^ signalling in vitro [166,167]. However, ICSs are less effective in patients with neutrophilic inflammation or corticosteroid resistance.

Adjunctive treatments can further modulate Ca^2+^-linked epithelial and ASM dysfunction. Macrolides, such as azithromycin and clarithromycin, reduce IL-13-induced goblet cell hyperplasia and epithelial apoptosis while normalising mitochondrial Ca^2+^ handling, indirectly improving epithelial integrity and mucociliary function [168,169,170]. Biologics targeting IL-4Rα, such as dupilumab, inhibit IL-13 signalling at its receptor, suppressing pathological Ca^2+^-mediated effects on both ASM and epithelium, providing a mechanism-based approach for selected COPD phenotypes with Th_2_-driven inflammation [1,171,172].

IL-13 drives Ca^2+^ dysregulation in ASM and airway epithelium, linking type 2 inflammation with bronchoconstriction, epithelial apoptosis, and impaired MCC. ICS remain the cornerstone for eosinophilic COPD [152], while macrolides and IL-13–targeted biologics offer mechanistic strategies to restore Ca^2+^-dependent ciliary function, reduce pathological mucus, and preserve airway integrity in patients with steroid-resistant or Th_2_-high phenotypes.

## 5. Cyclic Adenosine Monophosphate Signalling and Phosphodiesterases in Patients with COPD

Intracellular levels of cAMP are central to the regulation of inflammatory responses and mucociliary function in COPD. Under physiological conditions, elevated cAMP activates PKA, which inhibits the pro-inflammatory GTPase RhoA, suppressing nuclear factor kappa B signalling and reducing the production of cytokines and chemokines. In COPD, decreased cAMP concentrations, partly due to overexpression of PDEs, lead to diminished PKA activity. This disinhibition of RhoA contributes to persistent airway inflammation and impaired epithelial homeostasis [173].

Among PDE families, PDE4 isoforms, particularly PDE4A, PDE4B, and PDE4D, are upregulated in various immune cells and lung tissues of COPD patients. Alveolar macrophages and neutrophils show significantly higher mRNA levels of these subtypes compared to healthy controls, with PDE4A4 and PDE4B2 also upregulated in peripheral blood monocytes of smokers. Cigarette smoke exposure further increases PDE4 activity, accompanied by elevated PDE4A, PDE4B, and PDE4D expression in the airways [132,174].

Despite their therapeutic potential, the clinical use of PDE4 inhibitors is limited by adverse effects such as gastrointestinal disturbances and dizziness. To improve tolerability, subtype-selective PDE4 inhibitors targeting PDE4A–D are being developed to minimise systemic exposure. Inhaled PDE4 inhibitors, such as tanimilast, aim to enhance local efficacy, while dual PDE3/PDE4 inhibitors, such as ensifentrine, combine bronchodilatory and anti-inflammatory effects. Ensifentrine has demonstrated improvements in lung function and dyspnoea in phase III trials and may support MCC by increasing intracellular cAMP, thereby enhancing CBF and airway surface hydration [1,16,175,176,177].

PDE1A, a Ca^2+^/calmodulin-dependent PDE, plays a key role in ciliary regulation. Localised within the ciliary axoneme near the outer dynein arm, PDE1A modulates intracellular cAMP levels in a Ca^2+^-dependent manner, with its activity tightly regulated by [Ca^2+^]i, through calmodulin binding cells [151,153]. PDE1A inhibition has been associated with anti-inflammatory effects, including attenuation of IL-13 and MIP-1β expression in allergic airway inflammation [178,179,180].

In COPD, chronic cigarette smoke exposure has been reported to reduce PDE1A expression in bronchial epithelium and lung tissue [154]. While reduced PDE1A abundance could impair the fine-tuning of Ca^2+^–cAMP cross-talk in the axoneme, the consequent reduction in local cAMP hydrolysis may paradoxically increase cAMP availability and thereby partly support CBF as a compensatory mechanism.

**Figure 5:**
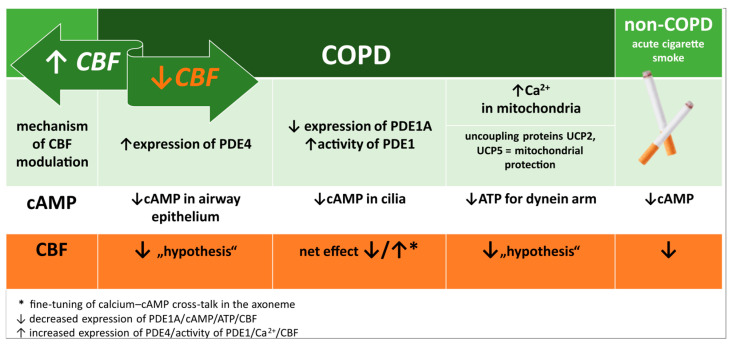
**Mechanisms of Ciliary Beat Frequency Regulation in COPD with Potential Pharmacological Approaches to Restore Ciliary Activity** (description can be seen in detail in the text). Note: PDE4—selective phosphodiesterase-4; PDE1A—selective phosphodiesterase-1A; PDE4i—selective phosphodiesterase-4 inhibitor; PDE3/4i—dual phosphodiesterase 3/4 inhibitor; PDE1Ai—selective phosphodiesterase-1A inhibitor; PDE4/1Ai—dual phosphodiesterase 4/1A inhibitor; cAMP—cyclic adenosine monophosphate [18,21,56,105,115,132,151,154,155,158,174,183,184].

On the other hand, oxidative stress may paradoxically increase PDE1 activity, lowering cAMP and amplifying pro-inflammatory signalling [155]. The interplay of reduced PDE1A expression and stress-induced activation results in variable effects on cAMP levels and ciliary function in COPD, depending on local oxidative and cellular conditions [154,181,182]. In other words, although PDE1A expression can be reduced in COPD, the residual enzyme may be hyperactivated, and together with upregulated PDE4 this leads to overall cAMP depletion at the cellular level. In COPD patients receiving LAMA therapy, intracellular Ca^2+^ signalling is further diminished, which may modulate PDE1A activity and cAMP levels, influencing the net effect on CBF and MCC. Consequently, the functional outcome for ciliary regulation depends on the balance between (i) loss of PDE1A expression, which may locally increase cAMP, (ii) stress-driven activation of remaining PDE1A and other PDE isoforms, reducing global cAMP, and (iii) the degree of Ca^2+^ dysregulation and mitochondrial dysfunction in the tissue. Together, these factors determine the net effect on CBF and MCC in COPD patients (Figure 5).

## 6. Results and Discussion

### 6.1. Impact of Tobacco Smoke on Respiratory Ciliary Beat Frequency

#### 6.1.1. Smokers Without COPD

In smokers without COPD, baseline cytosolic Ca^2+^ remains within the physiological range despite a reduction in Ca^2+^ influx [117]. The regulatory balance between Ca^2+^ and cAMP appears to be preserved, potentially through PDE1A activity [151], although evidence on PDE1A expression in this population is not yet conclusive. Acute cigarette smoke exposure, however, significantly lowers both baseline cytosolic Ca^2+^ and cAMP (Figure 5), leading to impaired ciliary beating and airway surface dehydration, partly mediated by reduced transepithelial chloride transport [18,117,183].

#### 6.1.2. Smokers with COPD

In smokers with COPD, Ca^2+^ homeostasis is profoundly disturbed. While baseline cytosolic Ca^2+^ may remain within the physiological range, endoplasmic reticulum Ca^2+^ release and store-operated influx are impaired, and these changes are accompanied by mitochondrial Ca^2+^ overload [105,115,117,118,184]. This overload promotes oxidative stress, mitochondrial apoptosis, and energy deficiency, thereby reducing ATP availability for dynein-driven motility (Figure 5). Structural consequences include ciliary shortening, reduced density, and disorientation [71,73,76,77]. In parallel, mucus becomes more viscous, and airway surface dehydration further compromises MCC [90,91,94].

At the signalling level, PDE1A expression in bronchial epithelium appears to be reduced, impairing physiological Ca^2+^–cAMP cross-talk [154]. However, PDE1A activity is paradoxically increased, accelerating cAMP degradation (Figure 5) [155]. Together with mitochondrial dysfunction, this may reduce ATP-derived cAMP availability, contributing to the reduced ciliary activity observed in several studies [15,71]. Acute cigarette smoke exposure can exacerbate this imbalance by causing a significant fall in cytosolic Ca^2+^ [117].

Nevertheless, evidence regarding CBF in COPD remains inconsistent. Some studies confirm reduced ciliary beating in COPD airways, whereas others report preserved or even increased CBF in nasal epithelial samples from smokers [18,117,185,186]. Methodological differences, including sample origin (bronchial vs. nasal epithelium), acute versus chronic smoke exposure, and the potential influence of systemic pharmacotherapy (e.g., PDE4 inhibitors), likely contribute to these discrepancies. From a clinical perspective, such variability underscores the importance of considering both disease severity and ongoing treatment when interpreting CBF data in COPD.

In smokers without COPD, transient smoke-induced reductions in Ca^2+^ and cAMP may slow ciliary motion but leave regulatory mechanisms largely intact. In smokers with COPD, however, mitochondrial Ca^2+^ overload, oxidative stress, and PDE-driven cAMP degradation create a setting of chronic energy and signalling deficits that compromise ciliary motility. Discrepancies across studies highlight the importance of patient stratification and the potential confounding role of pharmacotherapy when interpreting CBF data.

### 6.2. Impact of Pharmacotherapy on Respiratory Ciliary Beat Frequency

- **LAMA:** Exhibits a direct cilia-stimulatory effect, independent of classical second messengers (Ca^2+^, cAMP, PKA, purinergic signalling) [13].
- **LABA:** Enhances ciliary activity indirectly via the cAMP–PKA–dynein pathway [12,156,157].
- **LAMA + LABA**: Dual bronchodilator therapy may produce additive effects on CBF.
- **ICSs**: Generally neutral regarding ciliary activity, though they may influence calcium signalling in vitro. Clinical data suggest no significant impairment of MCC in COPD patients [166,167].
- **Triple therapy (LAMA + LABA + ICS)**: May enhance CBF due to LABA-mediated potentiation of ICS anti-inflammatory effects, including suppression of IL-13. However, ICS use in COPD is limited to patients with eosinophilic inflammation and carries an unfavourable side-effect profile. ICS may also upregulate β_2_-adrenergic receptors, thereby enhancing LABA-induced cAMP signalling and indirectly supporting CBF [162,187,188].
- **Selective PDE4 inhibitor, roflumilast**: By preventing cAMP degradation, promotes ciliary activity, though clinically relevant effects often require concurrent LABA administration [158]. Beyond its anti-inflammatory action, PDE4 inhibition reduces mucus gland hyperplasia and upregulates aquaporin-5 (AQP5), thereby facilitating airway hydration and normalising mucus viscosity [189]. AQP5 is among the most abundantly expressed lung aquaporins, with reduced expression linked to excessive mucus production and lung function decline in COPD [190,191,192,193,194]. This dual mechanism highlights PDE4 inhibitors as potential modulators of MCC, although gastrointestinal side effects remain a limiting factor for roflumilast use.

In COPD, impaired Ca^2+^ homeostasis, oxidative stress, and mucus hypersecretion converge to CBF and impair MCC. Current pharmacotherapy can modulate these dysfunctions: LAMAs exert direct cilia-stimulatory effects, LABAs enhance ciliary activity through cAMP–PKA–dynein signalling, and dual or triple therapy may provide additive or synergistic benefits. ICSs appear neutral on CBF but indirectly support ciliary function by suppressing IL-13–driven inflammation. PDE4 inhibitors, such as roflumilast, sustain intracellular cAMP and promote CBF, though their clinical utility is constrained by adverse effects.

### 6.3. Clinical Aspects

In clinical practice and research, nasal brushing is a widely used, non-invasive method for assessing MCC, as it can be performed rapidly and without anaesthesia. However, several limitations must be acknowledged:

- **Heterogeneity of COPD populations**
  COPD patients are often treated as a uniform group in studies, without consideration of pharmacotherapy. This methodological limitation may significantly affect interpretation of CBF results, particularly in studies using nasal samples [19,71,103,195], since pharmacological regimens influence ciliary function in distinct ways.
- **Nasal ciliary brush samples**
  ○ Patients should not be regarded as a homogenous cohort.
  ○ It is essential to differentiate between:
    ▪ *COPD patients without systemic therapy* (receiving only inhaled bronchodilators, primarily acting in the lower airways, with negligible influence on nasal cilia).
    ▪ *COPD patients with systemic therapy* (treated with PDE inhibitors, theophylline, macrolides, or systemic corticosteroids), in whom nasal ciliary activity may reflect systemic drug effects.
  ○ Importantly, expression of PDE isoforms differs between nasal and bronchial epithelium: while PDE4A is downregulated in nasal tissue [154], other PDE4 subtypes (PDE4B, 4C, 4D) are not, suggesting tissue-specific regulatory patterns that complicate extrapolation from nasal to bronchial samples.
- **Bronchial ciliary brush samples (via bronchoscopy)**
  Stratification by pharmacotherapy is equally critical when evaluating bronchial CBF:
  ○ *Dual therapy*: patients on LAMA + LABA.
  ○ *Triple inhaled therapy*: patients on LAMA + LABA + corticosteroids (representing the eosinophilic phenotype).
  ○ *Combination with systemic therapy*: patients receiving PDE inhibitors, theophylline, or macrolides in addition to inhaled treatment.

This level of stratification is crucial for reconciling discrepancies reported in the literature and for the precise interpretation of ex vivo CBF findings. It highlights the need for tailored pharmacological analyses when assessing mucociliary dynamics in COPD, thereby ensuring that drug-specific effects are appropriately recognised.

Clinical pharmacotherapy can influence ciliary function and must be accounted for in the interpretation of ex vivo CBF data in COPD. Nasal brush samples, in particular, may predominantly reflect the effects of systemic rather than inhaled therapies. Moreover, variations in PDE isoform expression (e.g., reduced PDE4A levels in nasal but not bronchial tissue) further complicate extrapolation. Consequently, careful stratification of patients by treatment regimen is imperative to prevent misinterpretation and to harmonise inconsistent findings across studies.

## 7. Limitations

- **LAMA:** While LAMA agents exhibit a direct cilia-stimulatory effect, they may simultaneously contribute to ASL dehydration, resulting in mucus thickening and an unfavourable periciliary environment for effective ciliary function [142]. However, at least one study demonstrated that tiotropium did not impair MCC while reducing HRV-induced mucin production, suggesting a clinically neutral effect on MCC [140]. Potential therapeutic targets to further restore ASL hydration include AQP5 channels or PDE4 enzyme.
- **LABA:** LABAs stimulate ciliary activity indirectly via the cAMP–PKA–dynein pathway. However, their efficacy depends on intact cAMP signalling, which may be compromised in COPD due to enhanced PDE activity. In addition, tolerance represents a clinical limitation; thus, LABAs are best used in combination with PDE4 inhibitors, or corticosteroids (restricted to the eosinophilic COPD phenotype). Sustained cAMP signalling is essential for long-term enhancement of CBF [16,132,158].
- **Airway epithelial remodelling and ciliary loss:** In advanced disease stages, MCC becomes ineffective due to shortened cilia or structural loss of ciliated cells, rendering pharmacological modulation of CBF insufficient [71,73,76,77].

These insights highlight the need to balance oxidative stress management and pharmacological modulation of ciliary activity in COPD by:

- Promoting a rational interpretation of CBF in clinical assessments, taking into account the modulatory effects of both inhaled and systemic therapies.
- Supporting the development of targeted treatments for COPD subtypes, particularly those characterised by neurogenic mucus hypersecretion and impaired MCC.
- Identifying novel strategies to optimise pharmacotherapy, not only to manage symptoms and prevent exacerbations but also to preserve or restore ciliary function as a key component of airway defence.
- Recognising that oxidative stress persists even in the absence of smoking, highlighting the potential for mitochondria-targeted therapies [196] as an emerging research avenue.

## 8. Emerging and Potential Cilia-Stimulatory Therapeutic Strategies and Clinical Implications

Recent insights into ciliary biology have reshaped our understanding of airway dysfunction in COPD, highlighting mucociliary clearance as a critical determinant of disease progression and treatment response. These findings have stimulated the search for therapies that go beyond bronchodilation, addressing the epithelial and ciliary components of airway pathology. As COPD encompasses distinct molecular and inflammatory endotypes, the development of phenotype-specific pharmacological interventions offers a promising avenue for restoring mucociliary function and improving clinical outcomes.

**A.**  **Patients with neutrophil phenotype of COPD**
  - **AQP5 channel modulators:** Contribute to airway hydration and improved mucus rheology, complementing MCC mechanisms [189,190,191,192,193,194].
  - **Mucus-targeted therapies:** MicroRNAs including miR-141, miR-92a, and circZNF652 have emerged as potential regulators of mucus production [197].
  - **Inhaled PDE4 inhibitor:** Tanimilast, currently in phase III clinical development for COPD and asthma, may sustain intracellular cAMP and enhance ciliary activity [16,152].
  - **Inhaled dual PDE3/4 selective inhibitor:** Ensifentrine represents another promising approach to enhance bronchodilation and MCC [16,159].
**B.**  **Patients with eosinophil phenotype of COPD**
  - **PDE1A inhibitors:** Elevate both cAMP and cGMP levels, increase CBF, and offer antioxidant and anti-inflammatory effects. Their benefits may be especially relevant in COPD patients with eosinophilia and comorbid neurodegenerative disorders such as dementia [16,151,153,198].
  - **Dual PDE4/PDE1A inhibitors:** Provide synergistic elevation of intracellular cyclic nucleotides with cilia-stimulatory, anti-inflammatory, and antioxidant effects, while improving airway hydration [199,200].
  - **Dupilumab:** A human IgG4 monoclonal antibody targeting the IL-4 receptor α subunit, inhibits both IL-4 and IL-13 signalling pathways. In COPD patients with an eosinophilic phenotype, it may reduce goblet cell metaplasia and mucus hypersecretion while preserving ciliary function [201].
  - **Chloride channel-targeted therapies:** In COPD, TMEM16A is pathologically upregulated. This increased expression promotes Ca^2+^-dependent mucus granule exocytosis, contributing to goblet cell hyperplasia and mucus hypersecretion. TMEM16A inhibitors represent a promising approach for patients with eosinophilic COPD phenotypes, where IL-13–driven TMEM16A induction contributes to mucus hypersecretion. By reducing MUC5AC expression and limiting goblet cell hyperplasia, these agents may alleviate mucus overproduction. Although our findings indicate that TMEM16A blockade can transiently lower CBF, this reduction did not fall below physiological thresholds, suggesting that the net impact on MCC may remain favourable in IL-13–dominated airway inflammation [202,203]. Further studies are warranted to determine whether TMEM16A inhibition can achieve an optimal balance between mucus suppression and preserved ciliary motility in clinical practice.
  - **T_2_R agonists:** Bitter taste receptors (T_2_Rs) represent a novel class of G protein–coupled receptors expressed in the airway epithelium and airway smooth muscle. On activation, these receptors respond to bacterial products by initiating Ca^2+^-triggered NO production, which both directly kills bacteria and enhances MCC. NO stimulates cGMP synthesis and activates protein kinase G, leading to phosphorylation of ciliary proteins and an increase in CBF [33,204,205]. In airway smooth muscle, T_2_R agonists induce relaxation and reduce airway tone, offering an alternative to conventional β_2_-agonists. Importantly, T_2_R activation also exerts anti-proliferative effects on airway smooth muscle, suggesting potential to mitigate airway remodelling, which has been difficult to address with currently available therapies [206]. In eosinophilic COPD phenotypes, T_2_R agonists therefore represent a promising therapeutic strategy to enhance MCC, restore airway patency, and attenuate structural changes in the airways.

While current COPD pharmacotherapy indirectly supports ciliary function, its success depends on the structural integrity and coordinated activity of cilia. Novel and adjunctive therapies aimed at enhancing mucociliary clearance particularly phenotype-tailored combinations targeting inflammation, oxidative stress, and mucus overproduction represent an important step towards personalised, function-oriented COPD management.

## 9. Future Perspectives

Future research should aim to integrate cilia-targeted interventions into comprehensive COPD management strategies that reflect individual inflammatory phenotypes. This requires not only the identification of reliable biomarkers of ciliary dysfunction but also the validation of non-invasive assessment tools, such as nasal sampling and digital high-speed video microscopy, to monitor treatment responses. Combining mechanistic insights of basic science with clinical observations will help clarify whether restoring ciliary function can translate into measurable improvements in disease control, exacerbation frequency, and quality of life. Collaborative efforts between translational researchers and clinicians will therefore be crucial in advancing cilia-focused precision medicine in COPD.

## 10. Conclusions

The preservation of effective MCC remains a cornerstone of airway defence in COPD. Current pharmacotherapy, particularly bronchodilators and corticosteroids, can support ciliary function, yet their efficacy ultimately depends on the structural integrity of the cilia. Emerging therapeutic approaches, including modulation of ion channels, PDE inhibition, and targeting of type 2 inflammation, may complement standard treatment by restoring airway hydration, mitigating oxidative stress, and preventing epithelial remodelling. Future research should focus on integrating cilia-preserving strategies into personalised COPD management, with the goal of achieving not only symptomatic improvement but also a measurable impact on disease progression.

## Data Availability

No new data were created or analyzed in this study.
